# Exploring laypeople’s epistemic beliefs about medicine – a factor-analytic survey study

**DOI:** 10.1186/1471-2458-12-759

**Published:** 2012-09-10

**Authors:** Dorothe Kienhues, Rainer Bromme

**Affiliations:** 1 Department of Psychology, University of Muenster, Fliednerstrasse 21, Muenster, 49149, Germany

## Abstract

**Background:**

The aim of this study was to develop an instrument to measure laypeople’s beliefs about the nature of medical knowledge and knowing (the EBAM). Such beliefs should be a target of increased research interest because they influence how people handle medical information, for example in shared decision making.

**Methods:**

An online survey was completed by 284 participants. Items assessed different aspects of laypeople’s epistemic beliefs about medicine and explicitly focused on the appearance of medical knowledge in everyday life and the evaluation of different sources as a way to justify knowledge.

**Results:**

Factor analysis yielded a five-factor solution for the instrument. Dimensions covered by the instrument are certainty of medical knowledge, credibility of medical textbooks, credibility of medical information on the Internet, justification of medical knowledge, and preliminarity of medical knowledge.

****Conclusion**s:**

Results indicate that laypeople have meaningful beliefs about the nature of medical knowledge and the trustworthiness of different sources. The instrument developed seems promising for measuring laypeople’s epistemic beliefs about medicine, which may help to increase patients’ compliance in medical decision making.

## Background

This article describes the rationale and empirical testing of an instrument to measure laypeople’s epistemic beliefs about medicine (EBAM).

Why are laypeople’s epistemic beliefs about medicine important and of interest for health psychology issues? For shared decision making and for patients’ treatment adherence, it is necessary for patients to understand the complexity and, at times, uncertainty of medical knowledge [1,2]. Imagine a male patient diagnosed with high cholesterol. His general practitioner has made clear that he should avoid eating butter and red meat and that it might be of value to take some medication to lower the cholesterol level. The patient will probably search for additional information on what to do on the Internet [3] (Google finds about 104.000.000 results for the keyword “cholesterol”). There, he will come across web pages with statements on the dangerousness as well as on the harmlessness of eating butter. He will find web pages that advertise a specific drug to lower cholesterol and Internet forums where patients describe (adventurous) alternative ways in how they control high cholesterol. In other words, he will be confronted with a plenitude of information and a multitude of opinions on a specific health-related problem. As a result, forming a conclusion about his future behavior will be quite difficult and the treatment of cholesterol may in his view be a complex and ill-structured problem (which it might also be in experts’ view [4]). The patient will have to undertake the task to assess which information is more important and valid than other information, how conflicts between various perspectives of knowledge can be solved, and how conflicting evidence could be integrated into a viable framework of personal understanding and decision making. He will have to cope with the fact that there are alternative treatments, differing interpretations of symptoms or even several interpretations of the underlying pathophysiological explanations of illnesses and risks. How the patient assesses the controversies (both on his own and in the next consultation with the doctor), in how far he accepts that people disagree on what is the best solution, how he reconciles his own ideas with those of experts and whether he takes advice from his doctor is influenced by the beliefs he holds about the nature of medical knowledge and knowing, called epistemic beliefs.

Of course, whether the patient will take advice from his doctor and in how far he is willing to participate in the decision making process, is not only influenced by epistemic beliefs but also by many other factors, e.g. the patients’ degree of anxiety, age or even their numeracy skills [5]. Furthermore, various characteristics of the doctor will play a role, e.g. his general consultation skills [6] but also in how far he appears to be trustworthy and competent [7] (and this evaluation of trustworthiness and competence might again be influenced by epistemic beliefs, see below).

Most problems patients are confronted with in medical decision making require knowledge and expertise which goes far beyond laypeople’s understanding. That is, patients depend on experts' explanations and advice and are not able to acquire the knowledge and skills which would be necessary to directly assess the experts' knowledge claims. However, patients will probably have a more general idea about what constitutes trustworthy and reliable knowledge claims, how one can obtain knowledge, or how to identify credible sources for (medical) information. That is, patients can refer to their epistemic beliefs when they need to reflect critically about medical information, evaluate what to believe, and which knowledge claim to support [8,9]. Epistemic beliefs are furthermore seen to function as an “apprehension structure” [10]: they allow for an anticipation of the knowledge to be learnt or to be dealt with, which includes for example an expectation of the complexity of a topic and of how much is already known about a topic.

As outlined above, medical problem solving often does not result in one clear solution, and the lack of relevant information for the problem-solving process is common [11,12]. Because many health-related problems are contentious and lack clear-cut solutions, they can be described as ill-structured problems, that is, “problems about which reasonable people reasonably disagree” [13]. Research in non-medical disciplines suggests that epistemic beliefs play an important role in dealing with such problems [8,13]. Therefore, assessing laypeople’s epistemic beliefs about medicine is important.

As a result, patients’ epistemic beliefs play an important role in medical decision making. They can be seen as a “must” for the exercise of patient rights, as they can guide their medical decision making even though patients do not have a conceptual understanding of the problem at hand. However, also in the case that patients prefer a rather paternalistic decision making model, their epistemic beliefs will still play a role insofar as that they may guide the choice of the expert to be trusted.

Therefore, laypeople’s epistemic beliefs about medicine should become a topic for research in health psychology. Psychological research on epistemic beliefs provides a profound background for this target.

### Research on epistemic beliefs

During the last two decades, epistemic beliefs have become a target of increased research interest in developmental and educational psychology [14-16]. Epistemology as one cornerstone of philosophy embodies questions about the processes by which human knowledge is justified as well as questions about the nature of knowledge [17]. This leads to the most often used definition of the construct epistemic beliefs which distinguishes between the nature of knowledge and the nature (or process) of knowing as the two core sets of concerns [18]. How one conceptualizes knowledge and how it changes over time are seen as aspects of the nature of knowledge, while considerations about where knowledge comes from and how to make justifications refer to beliefs about the nature of knowing. Epistemic beliefs range from a less advanced view to more advanced epistemologies and develop through life and from educational experiences [19,20]. Whereas a less advanced view includes beliefs such as that knowledge is certain and stable, either true or false, and can be handed down by an authority, a more advanced view is characterized by beliefs that knowledge is rather complex and relativistic, by accepting the uncertainty and changeability of truth, and acknowledging that knowledge is construed individually.

It is assumed that people possess both discipline-general and discipline-specific epistemic beliefs (beliefs about specific academic fields) concurrently [21-23]. Discipline-specific beliefs are presumed to play a predominant role when working on a discipline-specific problem, while discipline-general beliefs influence motivation and engagement [17,24].

For example, studies reveal the important role of epistemic beliefs in dealing with (scientific) controversies [25-27]. An exemplary study [26] investigated the influence of high school students’ epistemic understanding on the critical interpretation of a dual-position text. In this study, an initial test of epistemic understanding asked participants to indicate for pairs of contrasting statements whether they think that only one of the views described is right or whether both views could have some rightness and – depending on the response to this question (“if both could be right”) –whether one view could be better than the other. According to this initial test, participants were assigned to three groups of different epistemic positions: participants primarily holding a less advanced (seeing knowledge as absolute and either right or wrong), moderate (seeing knowledge as idiosyncratic, so that all positions are equally right), or more advanced view (seeing knowledge as derived from reason, so that some positions are more justified and sustainable than others). All participants read a scientific text about genetically modified food, introducing both the position in favor and against such food. After reading the text, participants were asked to write a conclusion to the text. Findings showed that students with more advanced and with moderate epistemic understanding reflected better on the inconclusive nature of the debate on transgenic food than students with less advanced beliefs.

### Epistemic beliefs about medicine

To date, epistemic beliefs about medicine have rarely been considered in psychological or medical research [28]. This is in sharp contrast to the assumed importance of such beliefs (outlined above). Evidence-based medicine comes along with the need for advanced epistemic beliefs [29,30]. Epistemic beliefs about medicine should play a quite prominent role in everyday life, not least because “the life of every individual is ripe with opportunities for applying health related knowledge” [31].

After a thorough review of the literature, few studies explicitly assessed beliefs about the nature of medical knowledge and knowing, and these studies did not deal with laypeople. In one study [29], conceptions of medical knowledge in an exploratory interview study on second-year medical students were considered. Results show that most students started medical school with rather simplistic beliefs, for example, stating that if uncertainty existed in some aspects of medical knowledge, this uncertainty would only be temporary. Within their first two years in medical school, most students changed their understanding of medical knowledge with regard to its certainty and considered medical knowledge open to change or adaptation. That is, the experience that medical knowledge less black or white than assumed led students to question their former way of thinking about medical knowledge.

Another study [32] attempted to elicit and analyze general practice trainees’ and trainers’ beliefs about medical knowledge and knowing in a focus-group approach. It concluded that people’s epistemic beliefs are probably influential in the solution of medical problems.

In sum, the nature of medical knowledge and knowing is not yet well researched, especially with regard to laypeople’s beliefs. One first attempt to assess laypeople’s epistemic beliefs about medicine is to develop an instrument to survey such beliefs. However, no such medicine-specific instrument exists. To date, research on laypeople’s views on knowledge about medicine has only considered laypeople’s subjective theories of illnesses [33]. Therefore, the objective of the study described in the following is to develop an instrument to survey laypeople’s epistemic beliefs about medicine. More specifically, we aimed for an instrument that assesses different aspects of laypeople’s epistemic beliefs about medicine and that explicitly focuses on the appearance of medical knowledge in everyday life. Items should especially consider that laypeople must rely on others to justify their beliefs and particularly take into account the trustworthiness of different sources.

## Methods

### Sample

In this study, 284 people (125 male, 158 female) completed the questionnaire. Participants’ average age was 20.79 years (*SD* = 3.83). Among the participants, 161 were in the final classes of German higher school education, while the other 123 were students of various subjects, predominately belonging to the subject areas of humanities and social sciences (30.1%), natural sciences (13.8%), and economics (12.2%). Because none of the participants’ studies focused on medical knowledge (e.g., medicine, pharmacy), they were assumed to be laypeople in this domain.

IRB approval for psychological survey studies is not (yet) common practice in Germany. In consequence, we did not apply for IRB approval. We did not collect data with identifying information and participation was voluntary.

### Materials: construction of the instrument

The EBAM was intended to extend and refine earlier work on discipline-specific measurement of epistemic beliefs to reach a medicine-specific instrument. We aimed for a questionnaire that explicitly focuses on the appearance of medical knowledge in everyday life. That is, it should consider such aspects of epistemic beliefs that are crucial for laypeople’s search and evaluation of competing sources of medical knowledge. In the first step, we scanned existing discipline-specific questionnaires and surveyed their dimensions. As a result, we selected several dimensions that should also be included in the first item sample of the EBAM from the instrument “Epistemological Beliefs Assessment for Physical Science” (EBAPS) [34]. These were the structure of scientific knowledge (whether knowledge is a coherent whole or a bunch of weakly connected pieces), real-life applicability (whether knowledge is applicable to real life or only applicable to restricted spheres) and evolving knowledge (whether knowledge is only mere opinion or set in stone). We left out the EBAPS dimensions “nature of knowing and learning” and “source of ability to learn”, as they focus on aspects of intelligence and learning and are therefore in our view [35] and the view of many other researchers [18,36] outside the construct of epistemic beliefs. We furthermore wished to consider the dimension certainty of knowledge, which is arguably the most agreed on dimension in conceptualizations of epistemic beliefs [37-39]. The Global Certainty Scale [40], which focuses on the fallibility of scientific knowledge, seemed to be a promising source of inspiration. This scale consists of seven items measuring in how far knowledge and theories in the soft and hard sciences are perceived as certain and unchangeable respectively as changing and fallible (a sample item for this scale is “Scientific theories can be proven false at any time” (reverse scored)).

In addition, the EBAM should specifically take into account the situation that a layperson has to manage a medical problem. Therefore, we included items on the credibility of different sources in which a layperson might search for medical information, namely the credibility of information on the Internet and the credibility of information in textbooks. We also added items to address a specific aspect of laypeople’s justification of medical knowledge: How should one decide on a medical treatment? Should one rely on current scientific research results or on the experiences of an affected patient or those of a general practitioner? These aspects are especially important with regard to laypeople’s epistemic beliefs: Because laypeople do not have substantial knowledge or experiences themselves, they have to rely on others to justify their beliefs. Furthermore, we added several items focusing on theory versus facts as well as on the connectedness, respectively isolatedness of medicine-specific knowledge to assess the structure or simplicity of medical knowledge.

All items collected were then rewritten, shortened, or modified so that they clearly focused on the discipline of medicine and laypeople’s understanding of it and could be answered on a Likert scale. The resulting preliminary version of the survey was carried out with a small number of individuals (six students) who were interviewed after answering each question to ensure that items were interpreted in a similar and intended way. After these interviews, minor revisions of the items were made. The final item pool for the EBAM consisted of 43 items that could be answered on a five-point Likert scale, ranging from “I totally disagree” to “I totally agree”. We administered additional items that surveyed several demographical variables (age, sex, subject, and length of study when applicable).

### Procedure

The survey software “EFS Survey” by Globalpark® was used to build a web-based survey. Participants were asked to participate in two different ways. Members of a German social networking website for students were invited to participate and students in their final year of school were asked to participate during an open day at the psychology department. Participation was voluntary.This procedure was chosen because it was economic and it ensured diversity and a heterogeneous sample. In both data collections, the introduction of the survey emphasized that all questions of the survey focus on a personal view of medical knowledge and medical information, and that there are no right or wrong answers. Participants could take as much time as they needed to answer all questions, that is, there was no time limit.

## Results

### Appropriateness of the data

Before conducting a factor analysis, some parameters were checked to ensure that the data were appropriate for factor analysis: A Kolmogorov-Smirnov-test revealed that data are normally distributed. Bartlett’s test of sphericity was statistically significant at *p* < .05, indicating that items were not coincidentally correlated. The measure of sampling adequacy was .72 for the initial sample, which can be interpreted as “middling” [41]. In sum, data were appropriate for factor analysis.

### Decisions on model-fitting method, rotation method and number of factors retained

An Exploratory Factor Analysis (EFA) was determined as the most appropriate form of analysis to uncover the underlying structure of the set of variables. Maximum Likelihood (ML) was chosen as the model-fitting method [42,43] and we decided for an oblique rotation method (direct oblimin rotation), because the statistical independence of epistemic dimensions in the sense of uncorrelated factors is questionable.

According to the scree plot, a five-factor solution was compared with a six-factor solution [43]. A comparison of the item loadings showed that the five-factor solution had the cleanest factor structure, which will therefore be reported in the following results.

### Factor solution

The five-factor solution was obtained by successively omitting items with no substantial factor loadings. Following the rule of thumb for a minimum loading of .32 for an item [44], a total of 17 items were removed. As a result, the final factor solution consisted of 24 items. ML factor analysis generates a chi-square goodness-of-fit test for the sample. Results underscored the goodness of fit (*χ*^*2*^ = 217.01; *df* = 166, *p* <. 01).

The first factor focused on the solvability of medical questions and the certainty or stability of medical knowledge. Therefore, it was called Certainty of Medical Knowledge. The factor consisted of seven items and yielded an acceptable Cronbach's α-value (α = .69). The dimension ranged from beliefs that there are or will be clear answers to medical problems to beliefs that there is no strong supporting evidence for medical knowledge. An exemplar item of this factor is: “Research in medicine has shown that there is one clear answer to most problems.”

The second factor consisted of four items which focused on the credibility of textbooks as a source of medical knowledge. The factor had a satisfactory α coefficient value (α = .73). This dimension ranged from the assumption that medical textbooks always provide credible information to the perception that the credibility of medical textbooks is always questionable and should therefore be critically scrutinized. An example item from this factor is: “Medical textbooks almost always contain reliable statements with regard to medical research.”

The third factor consisted of four items focusing on the credibility of the Internet as a source of knowledge. Cronbach's α was .74. Comparable to factor 2, this dimension ranged from the assumption that medical information on the Internet is always credible to the perception that the credibility of medical information on the Internet is always questionable and should therefore be critically scrutinized. An item example is: “One can almost always receive verified medical information on the Internet.”

The fourth factor consisted of four items. The α coefficient reached a satisfactory .61. Items belonging to this dimension took into account whether only people who have a specific disease or physicians who are experienced in treating a specific disease have true medical knowledge. The factor also assessed the applicability of medical research results. One of the items loading on this factor is: “People who suffer from a disease usually know better what to do than physicians.”

The fifth factor consisted of five items, focusing on the preliminarity, respectively Stability of Medical Knowledge. It yielded an acceptable Cronbach's α-value (α = .60). An item representing this factor is “Scientific theories in medicine, that I currently assume to be right, can be confuted in the future.”

Table 1 displays the pattern matrix for the reported five-factor solution.

**Table 1:** Pattern matrix for the five-factor solution obtained through exploratory factor analysis

	**Factor**				
	1	2	3	4	5
Research in medicine has shown that there is one clear answer to most problems.	.541				
If one has to decide between different therapy advices, one should only heed the physician’s advice.	.533				
If physicians address themselves to the investigation of a question, they will find the correct answer to almost all questions.	.507				
Medical knowledge is indefeasible.	.445				
If different physicians predict the progress of a person’s disease, they almost always agree.	.412				
In medicine, facts speak for themselves.	.386				
Some day medical researchers will be able to clear up all medical questions.	.355				
Medical textbooks almost always contain reliable statements with regard to medical research.		-.719			
Medical textbooks are a trustable source for gathering medical findings.		-.718			
One can almost always receive verified medical information from medical textbooks.		-.583			
One should almost always critically scrutinize medical information from textbooks.		.522			
One can almost always receive verified medical information from the Internet.			.766		
The Internet almost always contains reliable statements with regard to medical research.			.710		
The Internet is a trustable source for gathering medical findings.			.538		
One should almost always critically scrutinize medical information from the Internet.			-.486		
Finally one can only trust the medical advice of someone who has the (same) disease.				.663	
People who have a disease usually know better what to do than physicians.				.569	
The knowledge gained by medical research is mostly not applicable to everyday life. It only refers to idealized experiments in the laboratory.				.466	
In choosing a therapy one should only follow the advices of a general practitioner, even though the latest medical research potentially shows different results.				.421	
Scientific theories in medicine, that we currently assume to be right, can be confuted in the future.					.599
Even medical knowledge has to be revised over and over.					.563
The viewpoints in medical research change constantly.					.456
Theories in medicine can be confuted anytime.					.416
Even though medical research deals intensively with the origin of different diseases like for example cancer, it does not find one clearly correct explanation.					.374
Number of items per factor	7	4	4	4	5
Cronbach’s α	.69	.73	.74	.61	.60

Note: Pattern matrix, EFA, oblimin rotation, delta = 0, Maximum-Likelihood extraction. The factor analysis was conducted on the original item scores. For the calculation of sum scores for the five factors, several items need to be recoded (see section “Recoding of the items for further analyses”).

### Estimated stability of the factor solution

For an estimation of the stability of the factor structure, the FS parameter was calculated, which takes into account that the interpretation of factor loadings is dependent on sample size [45]. For the EBAM, FS was .91, indicating a good accord between the found factor structure and the “real” factor structure.

### Recoding of the items for further analyses

For the calculation of sum scores for the five factors, several items need to be recoded after administering the questionnaire. For factor 2 and factor 3, the forth item needs to be recoded. Furthermore, all items of factor 5 should be recoded. As a result, for factor 1 higher scores indicate a deeper belief in the certainty of medical knowledge and in the solvability of medical questions. For factor 2 and factor 3, higher scores indicate a deeper belief in the trustworthiness of medical textbooks respectively in the trustworthiness of medical information on the Internet. For factor 4, higher values mean that participants believe more that medical knowledge is better justified by (daily) experience than by medical research. For factor 5, higher values point to a stronger belief in the stability of medical knowledge.

## Discussion

For the purpose of this study, the most important result is that a meaningful five-factor solution was found for the EBAM. Cronbachs’s α was suitable for all factors. Results not only indicated that measuring laypeople’s epistemic beliefs about medicine is possible with a questionnaire, they also showed that laypeople have meaningful beliefs about the nature of medical knowledge and the trustworthiness of different sources.

The dimensions found are similar to those found in most popular epistemic questionnaires, because the EBAM takes into account aspects of the certainty and stability of knowledge, the justification for knowing, and the source of knowledge. The dimension Certainty of Medical Knowledge focuses on the extent that medical knowledge is fixed. The dimension Justification of Medical Knowledge taps how laypeople justify knowledge, e.g. by making use of authority and expertise (similar to Hofer’s dimension “justification for knowing” [37]). However, the EBAM thereby also considers that people also make use of patients who have a specific disease (as specialized “experts”) to justify knowledge. The two EBAM factors on sources of knowledge, factor 2 and factor 3, both explicitly focus on the credibility of sources. In contrast to other instruments on epistemic beliefs, the EBAM does not explicitly consider the self as a knowing person. In the context of the EBAM where a layperson deals with medical information, it is rather unrealistic that the layperson becomes a knower herself or himself in the sense of an active maker of meaning. Instead, a layperson will probably use different and more or less reliable sources to come to know. Therefore, the factor solution for the EBAM underlines the importance of considering the role of sources in laypeople’s understanding of medicine: due to the fact that laypeople only have limited medical knowledge on their own, they have to rely on others. For example they may gain information from medical textbooks (Credibility of Medical Textbooks) or the Internet (Credibility of Medical Information on the Internet). The factor Stability of Medical Knowledge focuses on how stable or variable medical knowledge is perceived to be.

This first study on the EBAM was successful at reducing the number of items of the EBAM. One of our aims was to minimize the number of items for further analyses. Results suggested that 17 items of the initial sample should be excluded in further analyses. The EBAM is a promising new measure of laypeople’s epistemic beliefs about medicine. In future research, the instrument could be improved in by adding new items to enhance the number of items per factor and the item to factor loadings, because some factors (factor 2, factor 3 and factor 4) could be represented by more items, and for these factors item to factor loadings could be higher (however, the measurement of epistemic beliefs is always a challenge and instruments usually do not show excellent psychometric values [46]). Furthermore, subsequent studies should aim for a replication of the factor structure across other, more heterogeneous samples, as the sample in this first study is rather homogenous with regard to age and education. In addition, it will be interesting to investigate whether the EBAM is able to differentiate between specific subgroups, e.g. between persons with low and substantial prior medical knowledge. A further topic for future research is how people’s medicine-specific epistemic beliefs guide their decision making when facing a health problem.

## Conclusions

The EBAM exemplarily refers to everyday aspects of living in a knowledge society, in which people must assess and make judgments on knowledge for which they are not an expert. Our results suggest that laypeople’s epistemic beliefs about medicine include more “classic” aspects of epistemic beliefs but especially aspects of the nature of knowing like the credibility of sources and how knowledge can be justified. In solving medical problems, laypeople not only have to rely on experts' explanations and advice, but they also have to cope with the fact that they are not able to acquire the knowledge and skills which would be necessary to assess the experts’ knowledge claims. A real understanding of most medical topics requires conceptual knowledge about medical, biological and chemical structures and processes and goes far beyond a non-expert’s understanding of medicine, biology and chemistry. That means that the question which source is credible and relevant becomes even more important [9]. The factors found for the EBAM exemplify this importance.

We hope that the EBAM will in the long run enhance an understanding of what laypeople think about the nature of medical knowledge and knowing, and how this influences their processing of medical information and medical decision making. Which views about the nature of medical knowledge and knowing are typically held by which people? Which views can doctors expect from their patients? How do people who hold advanced views and people who hold less advanced views differ in dealing with medical information? Considering a patient’s epistemic beliefs would be helpful for the doctor-patient-relationship. Patients holding different epistemic beliefs about medicine may deal with medical information differently and they also may be differently persuaded to initiate a certain therapy. The introduction in this article to laypeople’s epistemic beliefs about medicine and why they are important will hopefully call attention to the construct.

## Competing interests

The authors have no potential conflict of interest related to the subject of the paper.

## Authors' contributions

DK and RB contributed equally to the conception of the study and the acquisition of data. DK analyzed the data and drafted a first version of the manuscript. RB reviewed and edited this version and DK and RB worked on subsequent revisions of the manuscript and approved the final draft. Both authors have read and approved the final version of the manuscript.

## Pre-publication history

The pre-publication history for this paper can be accessed here:

http://www.biomedcentral.com/1471-2458/12/759/prepub
